# Measuring COVID-19 health literacy: validation of the COVID-19 HL questionnaire in Spain

**DOI:** 10.1186/s12955-022-02050-5

**Published:** 2022-09-27

**Authors:** María Falcón, Carmen Rodríguez-Blázquez, Martina Fernández-Gutiérrez, María Romay-Barja, Pilar Bas-Sarmiento, Maria João Forjaz

**Affiliations:** 1 Department of Legal and Forensic Medicine, Biomedical Research Institute (IMIB), University of Murcia, Murcia, Spain; 2 National Epidemiology Centre, Carlos III Health Institute, Madrid, Spain; 3 Institute of Research and Innovation in Biomedical Sciences of the Province of Cadiz (INIBICA), University Institute of Research in Social Sustainable Development (INDESS), University of Cadiz, Faculty of Nursing, Venus Street, 11207, Algeciras, Cadiz, Spain; 4 National Centre of Tropical Medicine, Carlos III Health Institute, Madrid, Spain; 5 National Epidemiology Centre, Carlos III Health Institute, Health Services Research on Chronic Patients Network (REDISSEC), Madrid, Spain

**Keywords:** COVID-19, Health literacy, Psychometrics, Public health, Health surveys

## Abstract

**Background:**

The COVID-19 pandemic has highlighted the importance of health literacy to make informed preventive decisions. A specific COVID-19 health literacy questionnaire (CHL-Q) is included in the COVID-19 Snapshot Monitoring WHO initiative to conduct behavioral insights studies related to COVID-19. The objective was to assess the psychometric properties of a Spanish version of the COVID-19 Health Literacy Questionnaire (CHL-Q).

**Methods:**

Data quality, acceptability, internal consistency, and construct and structural validity were analyzed. A Rasch analysis was also performed. This cross-sectional, observational study was conducted on the Spanish general population after the first wave of the pandemic and after the end of the general lockdown by an online survey agency. 1033 participants (inclusion criteria were being 18 years or older and living in Spain), was extracted from a panel of approximately 982,000 participants. The sampling was stratified matching the Spanish general population in terms of age, gender, and area of residence. The CHL-Q includes 9 items and assesses people's knowledge, motivation and competencies to access, understand, evaluate, and apply information about COVID-19 in order to make informed decisions.

**Results:**

CHL-Q index presented a mean of 33.89 (SD = 9.4), and good fit to the Rasch model (χ2(32) = 34.672, p = 0.342, person separation index = 0.77), with ordered thresholds, unidimensionality, item local independence, and no item bias by sex, age or education level. The CHL-Q showed significant different scores by level of education, experience of infection, confusion related to COVID-19 information and adherence to preventive measures. We found a statistically significant correlation between the CHL-Q index and the total number of preventive measures adopted, COVID-19 knowledge, and information seeking behaviour. The Cronbach´s alpha was 0.87 and the item total corrected correlation, 0.49–0.68.

**Conclusions:**

The Spanish version of CHL-Q is a short, adequate, and reliable instrument to measure COVID-19 related health literacy in the Spanish general population. Measuring the CHL in the population can be useful to evaluate whether public authorities, media and the medical and scientific community have been able to reach the population to offer the information in the terms they need it.

**Supplementary Information:**

The online version contains supplementary material available at 10.1186/s12955-022-02050-5.

## Background

The COVID-19 pandemic due to the coronavirus SARS-CoV-2 represents one of the most significant public health crises that Spain and the world has faced in modern history. By May 2021, Spain had 3,586,333 confirmed COVID-19 cases, of which 340,130 required hospitalization and 79,100 had died [1].

The Spanish government and authorities all around the world have implemented different measures to contain the spread of the infection, ensure low fatality rate and minimize the socioeconomic impact of the pandemic [2, 3]. Current public health strategies are mainly based in changing personal lifestyles and are characterized by the central role of individuals’ adherence to recommended preventive measures. Politicians and public health advisors have called citizens to action asking for individual’s responsibility and solidarity [4].

To properly follow public health messages, people need to find accurate information, and understand and apply it in everyday life. In essence, we are talking about Health Literacy (HL), defined as the individual’s knowledge, motivation and competences to access, understand, appraise and apply health information in order to make appropriate health decisions [5]. Relevant information related to COVID-19 is varied, changes over time and may be complex, including diverse aspects such as transmission modes, symptoms, testing, preventive measures, quarantine recommendations and restriction measures, among others. This information is widely available through mass media, social media and many other online and offline sources. Besides that, “information consumers” not only have to deal with information overload, but also with misinformation [6].

From the beginning of the outbreak, authors have emphasized that HL plays an important role in pandemic control, and the need to take it into consideration in public health messages to reach everybody in the fight against the virus [7–9]. In fact, COVID-19 pandemic has highlighted that poor health literacy is a globally underestimated public health problem [4]. In addition, studies to date suggest a positive association between HL, COVID-19 related knowledge, and preventive behaviors [10–14]. A recent review on HL related to coronavirus outbreaks highlighted the need of developing and validating tools to assess pandemic-related HL [15].

The WHO has included a specific COVID-19 health literacy questionnaire (CHL-Q) in the COVID-19 Snapshot MOnitoring (COSMO) initiative, developed to conduct behavioural insights studies related to COVID-19 [16]. This questionnaire is based in a well-known generic instrument, the European Health Literacy Survey Questionnaire (HLS-EU-Q), developed to measure HL in a comprehensive way [5]. Following the HLS-EU-Q core model, the CHL-Q focuses on analysing the key processes of accessing, understanding, appraising and applying COVID-19 health related information. The CHL-Q is a subjective measure of health literacy, reflecting the interactive or relational nature of health literacy by assessing the fit of personal competences with contextual demands [5]. The aim of this study was to test the psychometric properties of a Spanish version of the CHL-Q, using Classic Test Theory and Rasch measurement analysis, as recommended for validation of rating scales [16].

## Methods

### Study design and settings

This study is part of a larger project, the COSMO-SPAIN Project, aimed to inform COVID-19 outbreak response measures, including policies, interventions and communications. The results have been shared with the Spanish health authorities and are publicly available (https://portalcne.isciii.es/cosmo-spain/).

A cross-sectional, observational study was conducted on the Spanish general population from 27 July to 3 August 2020 by an online survey agency. The sample, composed of 1033 participants, was extracted from a panel of approximately 982,000 participants. The sampling was stratified matching the Spanish general population in terms of age, gender, and area of residence. Inclusion criteria were being 18 years or older and living in Spain.

Data were collected after the first wave of the pandemic and after the end of the general lockdown (21st June 2020). At the time of the study the epidemiological situation in Spain had improved, and the accumulated incidence was 37.9 cases by 100,000 inhabitants [17]. The use of face masks was mandatory for people aged 6 years or older in enclosed spaces and outdoors. The government opened all internal borders among autonomous communities as well as international travel restrictions with other European Union countries and the United Kingdom. Other restrictions related to mass gatherings and closure of public spaces were handled by each autonomous community independently.

### Measurements

#### Sociodemographic characteristics

Participants were asked about their gender, age, education (incomplete primary; primary education; secondary education; high school education, and university), area of residence (2000 to 50.000; 50.000 to 400.000; > 400.000 inhabitants) and employment situation.

#### COVID-19 health literacy (CHL-Q)

CHL-Q items included in the COSMO-WHO survey tool [18], originally in English, were translated by professional translators and adapted by the COSMO-SPAIN team.

The original HLS-EU-Q, which assess generic HL, consists in 47 items, but shorter versions have been developed and tested in several languages and contexts [19–27]. In Spain, a short version was found to be an adequate and valid tool to measure the level of generic HL in the population [19].

The CHL-Q assesses people's knowledge, motivation and competencies to access, understand, evaluate, and apply information about COVID-19 in order to make informed decisions to prevent the disease. Includes a general question “How easy or difficult is it for you to…?” followed by 9 specific tasks, for which participants rate their perceived difficulty on a four-category Likert scale very difficult (1), difficult (2), easy (3) and very easy (4).

In accordance with the original questionnaire, the CHL-Q index was standardized from 0 to 50 [index = (mean – 1) × (50/3)], using the mean of all items for each respondent.

#### Other variables

We addressed COVID-19 infection by asking participants “To your knowledge, are you, or have you been, infected with COVID-19?”, with yes/no response options. In order to assess information-seeking behaviour, we asked the respondents about how often they used 8 common sources of information (TV news, TV and radio magazines, government press briefings, newspapers, social networks, internet, government website and WHO reports) to stay informed about the coronavirus, answered in a scale from 1 (never) to 5 (very often).

COVID-19 knowledge was measured by the degree of agreement with 12 correct and incorrect statements about COVID-19 (Supplementary file 1). The items were developed according to health authorities’ guidelines at the time of the survey, and included symptoms, transmissibility and face mask use (e.g. people who do not have fever can be contagious; the coronavirus is spread by droplets when coughing/talking; face masks must cover mouth and nose). The total number of correct answers composed the COVID-19 knowledge score (0–12).

To assess confusion related to COVID-19 information we asked the participants: “Have you encountered information on the novel coronavirus where you found it hard to decide whether it was right or wrong?” (yes/no).

Adherence to recommended preventive behaviours was assessed by 8 items regarding basic protective measures recommended at that time by the health authorities, “During the last 7 days, which of the following measures have you taken to prevent infection from COVID-19?” (yes/no). The total number of preventive measures taken by each participant composed the preventive adherence score, ranging from 0 to 8.

### Data analysis

CHL-Q index data was normally distributed, and thus, parametric statistics were applied. Continuous variables were expressed as central tendency measures (means, medians), measure of dispersion (standard deviation—SD, range), and the categorical variables were expressed by frequencies and percentages. Bivariate descriptive analyses were performed on the continuous variables by Spearman's correlation. To test the association between variables, for independent samples, ANOVA tests were used.

The psychometric properties of the CHL-Q were explored using Rasch analysis and classical test theory (CTT): data quality and acceptability, construct (structural and hypotheses testing) validity, and reliability (internal consistency) [28].

For data quality and acceptability, the mean, median, SD, range of observed vs. theoretical values, skewness (criterion: − 1 to + 1), floor and ceiling effects (≤ 15%) of the CHL-Q items and index were calculated [29]. Internal consistency of the CHL-Q was examined by computing by Cronbach’s α coefficient (≥ 0.70), item-total corrected correlations (r ≥ 0.40), inter-item correlations and the item homogeneity index (> 0.30) [30].

The corrected item-total correlation (≥ 0.20), for structural validity, and the inter-correlation of CHL-Q, for internal validity, using Spearman’s rank correlation coefficients were calculated.

Discriminative or known-groups validity were explored by calculating the differences in CHL-Q scores in the sample grouped by age, sex, education level, employment status, infection status, adherence to preventive measures and confusion related to COVID-19 information. Lower CHL-Q index scores were expected for older [5], those with lower education level [31], feel confused about COVID-19 information [32] and had been infected [33, 34].

Convergent validity was calculated using the Spearman’s rank correlation coefficient between the CHL-Q index and age, information seeking frequency, COVID-19 knowledge score and number of preventive measures taken. Our hypothesis was a low correlation of the CHL-Q index with age [5] and a moderate one with knowledge about COVID-19 [13], number of preventive measures [13, 14] and frequency of information seeking [35].

The Rasch model purports that the probability of a given response to an item is a function of the person’s capability (or level of health literacy) and item difficulty (or degree of the construct measured by the item), expressed in logits in an interval-level scale [36]. Fit to the Rasch model is obtained when there is a non-significant interaction chi-square difference between the observed data and the Rasch model [37]. Since a large sample size might lead to signalling small model deviations as significant, and to unnecessary model modifications, a random sample of 300 was used. This sample size provides stable calibrations independently of the targeting [38]. Item and person fit residuals are expected to follow a normal distribution with mean of 1 and SD of 0, and fall within the -2.5 to 2.5 interval. Reliability is assessed through the Personal Separation Index (PSI), interpreted similarity to Cronbach’s alpha. Low correlations (< 0.30 of the average correlation) between item residuals indicate item local independency, i.e., that the response to one item do not lead to the response to another item. Unidimensionality was ascertained by a principal component analysis of the residuals and comparison through t-tests; the lower bond of the associated binomial 95% confidence interval should be ≤ 0.05. Item thresholds, or the point of equal response probability between two adjacent response categories, are expected to be ordered. For Differential Item Functioning (DIF), analyses of variance were performed by the following groups of persons: age (lower than the median 46 vs. 46 + years), sex, and education level (low: up to 14 years old; medium: secondary or professional training; high: university).

Analysis was performed using IBM SPSS Statistics version 22 (IBM, Armonk, NY). Rash analysis was performed iteratively, using RUMM2030 software.

## Results

### Sample characteristics

The average age of the participants was 45.7 (SD = 14.6; range 18–89) years and 49.7% were women (Table 1). The proportion of the sample living in a rural area was 35.1%. Most of the participants had a medium–high education level (75.7%) and were employed (56.5%).

**Table 1:** Characteristics of the sample

	N (%)
Gender	
Female	513 (49.7%)
Male	519 (50.3%)
Age groups (years)	
18–29	166 (16.1%)
30–44	308 (29.8%)
45–60	344 (33.3%)
> 60	214 (20.7%)
Education	
Incomplete primary	17 (1,6%)
Primary	28 (2,7%)
Secondary	206 (19.9%)
High school	318 (30.8%)
University	464 (44.9%)
Area of residence	
2000 to 50,000 inhabitants	362 (35.07%)
50.000–400,000 inhabitants	402 (38.95%)
> 400.000 inhabitants	268 (25.96%)
Employment situation	
Working	584 (56.5%)
Student	70 (6.8%)
Domestic care	78 (7.6%)
Retired/pensioner	154 (14.9%)
Long-term unemployed	91 (8.8%)
Unemployed COVID or ERTE	56 (5.4%)

ERTE: Spanish temporary employment regulation due to COVID-19. In Spanish, “expediente de regulación temporal de empleo”; HL: Health literacy; SD: standard deviation

### Psychometric properties

The CHL-Q index was computable for 1032 (99.9%) participants. Mean score of the index was 33.89 (SD = 9.40; range: 0–50). Detailed statistics for the CHL-Q index and the individual items are described in Table 2. All items showed closeness of mean to the median, covered the full range of scores (1–4), and had no skewness or floor effect. Ceiling effect was present in all CHL-Q items. The scale showed a Cronbach´s alpha of 0.87 and the item total corrected correlation was 0.49–0.68 (Table 2). CHL-Q items correlated between them from 0.255 (item 6-item 8) to 0.749 (item 1-item 5), with an item homogeneity index of 0.43.

**Table 2:** Data quality, acceptability and internal consistency of COVID-19 health literacy questionnaire (CHL-Q)

CHL-Q items	Mean	Median	SD	Skewness	Min	Max	Floor effect %	Ceiling effect %	Item-total corrected correlation	Cronbach’s alpha if item deleted
1. Find information about symptoms of COVID-19	3.24	3.00	0.76	− 0.81	1	4	2.6	41.1	0.60	0.86
2. Find out about political decisions on restrictions related to coronavirus/COVID-19	3.11	3.00	0.76	− 0.58	1	4	2.9	32.0	0.66	0.85
3. Find out what to do in case you suspect you have COVID-19	3.05	3.00	0.78	− 0.57	1	4	3.8	29.5	0.61	0.85
4. Understand what authorities say about the coronavirus/ COVID-19	2.95	3.00	0.89	− 0.52	1	4	7.3	30.0	0.64	0.85
5. Understand what authorities say about coronavirus/ COVID-19 restrictions and recommendations	3.14	3.00	0.80	− 0.68	1	4	3.6	36.5	0.68	0.85
6. Judge if the information about coronavirus/COVID-19 in the media is reliable	2.58	3.00	0.92	− 0.06	1	4	12.8	17.2	0.49	0.87
7. Judge when you need to go to the doctor for non-COVID-19 related problems	2.91	3.00	0.82	− 0.29	1	4	4.2	25.9	0.58	0.86
8. Follow the recommendations on how to protect yourself from coronavirus/COVID-19	3.27	3.00	0.72	− 0.77	1	4	2.0	40.9	0.64	0.85
9. Decide when to stay at home from social activities/ work/school, and when not to	3.04	3.00	0.80	− 0.51	1	4	3.7	30.9	0.58	0.86
CHL-Q index	33.90	33.33	9.40	− 0.42	0	50	0.6	6.2	–	0.87*

^*^Cronbach’s alpha the full scale

Convergent and discriminative validity data of the CHL-Q are summarized in Table 3. There were not significant differences in CHL-Q index by gender, age, area of residence, and employment situation groups. The CHL-Q index showed significant different scores by level of education (p = 0.009), with higher scores in participants with university degree; by experience of infection (p = 0.01), with lower CHL-Q scores in participants who reported having been infected; by confusion (p < 0.001), with lower CHL-Q scores in those who had difficulties to decide if the information on COVID-19 was correct; and by adherence to preventive measures, with lower CHL-Q scores in those who reported noncompliance with almost all the preventive measures, except for hand washing and staying at home when sick.

**Table 3:** Convergent and discriminative validity of COVID-19 health literacy questionnaire (CHL-Q)

		n (%)	CHLI mean (SD)	p^a^	Correlation with CHL-Q index^b^
**Education**				0.002	
Incomplete primary		17 (1.6)	30.32 (12.89)		
Primary		28 (2.7)	27.72 (9.57)		
Secondary		206 (19.9)	33.48 (8.85)		
High school		318 (30.8)	34.26 (9.41)		
University		464 (44.9)	34.33 (9.35)		
**COVID-19 related variables**					
COVID-19 experience	Yes	54 (5.23)	30.03 (10.14)	0.010	
	No	978 (94.76)	34.11 (9.31)		
Confusion	Yes	583 (56.4)	33.12 (8.7)	< 0.002	
	No	449 (43.6)	34.91(10.15)		
**Preventive measures**					0.12**
Wearing face masks	Yes	946 (91.6)	34.32 (9.20)	< 0.001	
	No	87 (8.4)	29.24 (10.35)		
Washing hands	Yes	931 (90.1)	34.04 (9.48)	0.126	
	No	102 (9.9)	32.59 (8.56)		
Hydroalcoholic gel use	Yes	894 (86.5)	34.30 (9.24)	0.004	
	No	139 (13.5)	31.28 (10.04)		
Not meeting with relatives/friends	Yes	587 (56.8)	34.40 (9.25)	0.009	
	No	446 (43.2)	33.22 (8.57)		
Stay at home in case of symptoms	Yes	337 (32.6)	33.62 (10.02)	0.712	
	No	696 (67.4)	34.03 (9.09)		
Keeping the distance	Yes	875 (84.7)	34.34 (9.29)	< 0.001	
	No	158 (15.3)	31.44 (9.66)		
Disinfecting surfaces	Yes	569 (55.1)	34.52 (9.60)	0.013	
	No	464 (44.9)	33.12 (9.10)		
Avoiding public transport	Yes	370 (35.8)	34.49 (9.37)	0.005	
	No	663 (64.2)	32.82 (9.36)		
		**Mean (SD)**			
**COVID-19 knowledge**		9.33 (1.68)			0.15**
**Frequency of information seeking**					
Tv news		3.76 (1.18)			0.21**
Tv and radio magazines		3.04 (1.27)			0.13**
Government press briefings		2.91 (1.20)			0.18**
Newspapers		3.21 (1.27)			0.19**
Social Networks		3.00 (1.35)			0.04
Internet		3.58 (1.20)			0.17**
Government website		2.56 (1.30)			0.15**
WHO reports		2.72 (1.22)			0.17**

CHL-Q: COVID-19 health literacy questionnaire.^a^ANOVA; ^b^Pearson correlation. **p < 0.001

CHL-Q index correlation coefficients were r = 0.12 (p < 0.001) with the total number of preventive measures adopted, r = 0.15 (p < 0.001) with COVID-19 knowledge, and r = 0.13–0.21 (p < 0.001) with frequency of information seeking in all the media sources included in the survey, except for social networks r = 0.04 (p = 0.214).

The first Rasch analysis with a random sample of 300 showed a good fit to the Rasch model, χ^2^(36) = 53.616, p = 0.0297, PSI = 0.83. However, item 6 displayed a high fit residual, 3.386, with a significant chi-square value. This problem persisted when using a different random sample of 300.

**Fig. 1:**
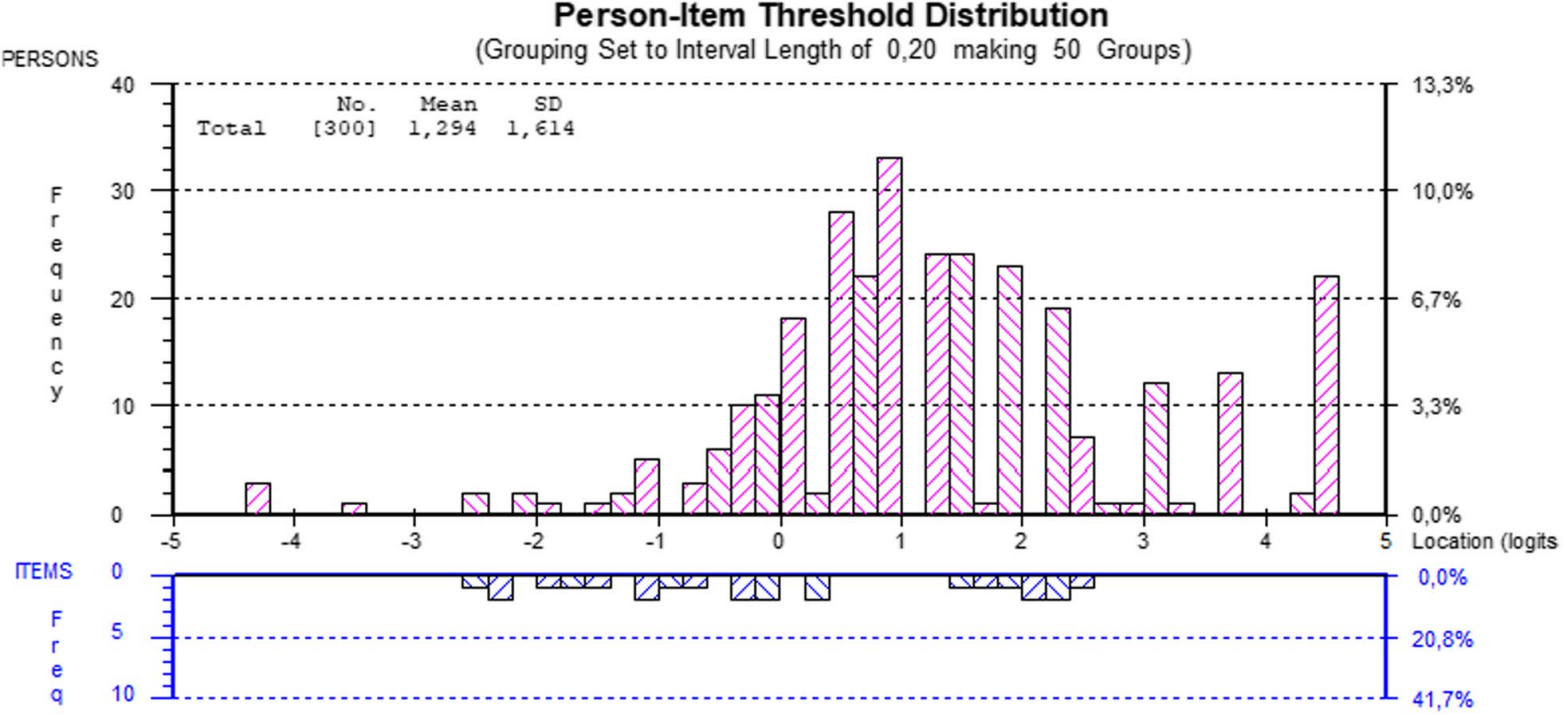
Person-item threshold distribution

After deletion of item 6, there was a goof fit to the Rasch model, χ^2^(32) = 34.672, p = 0.342, PSI = 0.77 (Table 4), ordered thresholds, unidimensionality, item local independence, and no DIF by sex, age, or education level. Table 4 presents the individual item fit. The third threshold of item 7 represents the highest literacy level, whereas the first thresholds of items 1 and 3 correspond to the lowest. Figure 1 displays the person-item threshold distribution, showing absence of floor or ceiling effect and item thresholds representing the most part of the person distribution, although less in the distribution extremes.

**Table 4:** Goodness of fit to the Rasch Model and individual item fit of the COVID-19 health literacy questionnaire (CHL-Q)

Attribute		Criteria	CHL-Q index
Item fit residual	Mean	0	0.139
	SD	1	0.506
Person fit residual	Mean	0	− 0.556
	SD	1	1.632
Item-trait	χ^2^(df)	Low	34.67 (32)
interaction	*p *value	NS	0.342
PSI		> 0.70	0.775
Unidimensionality	Independent t-tests	< 5%	6.67%
	CI 95% Binomial	*	0.042–0.091

Item	Location	Standard error	Fit residual	Chi-square (df = 4)	*p *value
1. Find information about symptoms of COVID-19	− 0.499	0.1	0.393	0.913	0.923
2. Find out about political decisions on restrictions related to coronavirus/COVID-19	− 0.177	0.100	− 0.150	3.219	0.522
3. Find out what to do in case you suspect you have COVID-19	− 0.176	0.101	− 0.429	3.304	0.508
4. Understand what authorities say about the coronavirus/ COVID-19	0.600	0.088	0.357	10.480	0.033
5. Understand what authorities say about coronavirus/ COVID-19 restrictions and recommendations	− 0.005	0.093	0.001	2.637	0.620
7. Judge when you need to go to the doctor for non-COVID-19 related problems	0.354	0.094	0.771	1.886	0.757
8. Follow the recommendations on how to protect yourself from coronavirus/COVID-19	− 0.422	0.102	− 0.565	10.325	0.035
9. Decide when to stay at home from social activities/ work/school, and when not to	0.325	0.089	0.737	1.910	0.752

^*^ Lower bond should be ≤ 0.05. SD: Standard Deviation; NS: non-significant. PSI: Personal Separation Index; CHL-Q: COVID-19 health literacy questionnaire

## Discussion

The Spanish version of the CHL-Q is based in that proposed in the WHO COSMO questionnaire to conduct behavioural insights studies related to COVID-19. The validation of a specific COVID-19 HL tool is essential for monitoring the levels of health literacy related to COVID-19 in population surveys and tailoring the pandemic-prevention strategies to the population’s level of COVID-19 related HL. The CHL-Q is a short and simple measure, based on previous HL scales, which supports its content validity [5].

The results show that the CHL-Q in Spanish is reliable, and although the questionnaire contains items related to different competencies in information management, it is unidimensional. Furthermore, its convergent and discriminant validity have been confirmed.

Most data quality and acceptability parameters were within the standard limits. However, all items showed a marked ceiling effect, indicating that most participants perceived very easy to find, understand, appraise, and apply the COVID-19-related information. This was also found in the original HLS-EU-Q, which measures generic HL, indicating that the indices are more sensitive for lower than for higher health literacy scores [39]. The internal consistency and reliability of the tool was satisfactory, with all items showing high item-total corrected correlation. These results are similar to the different versions of the HLS-EU-Q that have been previously validated [26, 27]. However, item 6 showed the lowest item-total corrected correlation, and Rasch analysis suggests it measures a different construct. After deletion of this item, reliability remained high. Further studies are needed to confirm the pertinence of this item.

The Rasch analysis showed that the scale fits into a unifactorial model. The original questionnaire of generic HL comprises 47 items grouped in four dimensions across three domains [39]. However, studies analysing the factor structure of the shorter versions show contradictory results [19, 21–27]. In this sense, some studies have found a unidimensional structure [22, 23, 25] while others have identified three or four factors [21, 22, 26]. Two studies have applied Rasch analysis to determine the structure of the 12-item and 16-item scale [23, 25], with findings that confirm the unidimensionality of the HLS-EU-Q in a similar way to the CHL-Q in the present study. Rasch analysis also showed that the response scale is adequate.

As expected, CHL-Q scores were higher in those with higher education level, but no relationship was found with sex, age, employment, or rural/urban residence. Studies exploring the association between generic HL and sociodemographic variables have shown conflicting results, being education level, the variable most consistently linked to HL [5, 22, 23]. The absence of DIF by education level indicates that the observed differences are not due to a bias. As in Spain, association between COVID-19 related HL and sex or age was not found in Germany, which may be due to the efforts made by authorities of both countries to reach the general population, making it easy to access, understand, appraise, and apply coronavirus health information in everyday life [32].

We have found lower CHL-Q scores in those who reported having been infected. Low levels of HL have been proposed as a risk factor for COVID-19 infection [34]. Those who have encountered hard to decide if information on the novel coronavirus was right or wrong have scored significantly lower in the CHL-Q. This is in accordance with the results reported in Germany [32]. Also, in Australia there was a markedly higher endorsement of some common misinformation statements about COVID-19 in people with lower HL [10]. The WHO has already addressed this issue stating that “we’re not just fighting an epidemic; we’re fighting an infodemic” [6].

Regarding convergent validity, the CHL-Q index correlated with the number of preventive measures adopted, the general knowledge on COVID-19, and the frequency of seeking information, although the correlation coefficients were low. The correlation between CHL-Q index and individual’s preventive behaviours is consistent with previous studies reporting a positive association between HL and the adoption of protective measures among chronic patients [14], adolescents [13] and university students [12]. The reported adherence to protective measures is very high in Spain (https://portalcne.isciii.es/cosmo-spain/), where restriction measures and public health messages have been widely spread. Likewise, the relationship between CHL-Q scores and knowledge related to COVID-19 is consistent with recent studies that show better COVID-19 related knowledge in those with better generic HL [10, 11, 13, 14]. According with previous research [35], individuals with higher health literacy were more active information seekers.

The main limitation of this study is that the survey was only administered online (adapted to computer or smartphone). This way, people with lower educative level are under-represented in the sample, which may introduce some bias. However, the study used a nationally representative sample in terms of age, gender, and area of residence. In addition, COVID-19 cases were based on a self-reported diagnosis. Finally, we did not include a previously validated HL questionnaire, which would allow to study criterion validity. Further studies should assess CHL-Q validity with cross-national samples and analyse test–retest reliability.

## Conclusion

The CHL-Q is a short, adequate, and reliable instrument and provides valid data to measure the level of COVID-19 related HL in Spanish general population. After deleting one item, it shows good measurement properties according to the Rasch model, with unidimensionality, adequate response scale, item local independency and no item bias by age, sex, or education level.

An adequate health literacy is essential to cope with the COVID-19 pandemic as it helps people to acquire and use credible health related knowledge and adopt protective behaviors. In the current situation, measuring the CHL in the population is useful to evaluate whether the public authorities, the media and the medical and scientific community have been able to reach the population to offer the information in the terms in which the citizens need it. The results indicate that low levels of Health Literacy are a risk factor for COVID-19 infection.

## Supplementary Information

**Additional file 1.** COVID-19 knowledge.

## Data Availability

The datasets generated and/or analysed during the current study are not publicly available due to the funding institution have to authorized first but are available from the corresponding author on reasonable request.

## Abbreviations

CHL-Q: COVID-19 health literacy questionnaire
COSMO: The COVID-19 snapshot monitoring initiative
CTT: Classical test theory
DIF: Differential item functioning
HL: Health literacy
HLS-EU-Q: The European health literacy survey questionnaire
ISCIII: Carlos III health institute
PSI: Personal separation index
RMSEA: Root mean squared error of approximation
SD: Standard deviation
WHO: World health organization
