# Supplementary material for: Measuring COVID-19 health literacy: validation of the COVID-19 HL questionnaire in Spain
Source: Health Qual Life Outcomes. 2022 Sep 27;20:138. doi: 10.1186/s12955-022-02050-5 (PMC9514704; doi:10.1186/s12955-022-02050-5)
Supplement: Supplementary file 1 — Additional file 1. COVID-19 knowledge. [file 12955_2022_2050_MOESM1_ESM.docx]

| **Indicate the most frequent forms of contagion of the coronavirus** | **Correct answer** |
| --- | --- |
| Drops when coughing/talking | X |
| Contaminated surface | X |
| Physical contact with someone infected | X |
| By blood transfusion |  |
| From an insect bite |  |
| Contact with pets (dog, cat, others) |  |
| **According to you, the mask… (Mark the options that you consider)** |  |
| It is used to avoid infecting others. | X |
| It is used to protect itself from being infected | X |
| Hands should be washed before and after use | X |
| You have to cover your nose and mouth | X |
| It should be touched only by the ear tape | X |
| Must be removed for coughing or sneezing |  |

**Supplementary File 1. COVID-19 knowledge**
